# Developing a New Marker of Dynamic Hyperinflation in Patients with Obstructive Airway Disease - an observational study

**DOI:** 10.1038/s41598-019-43893-1

**Published:** 2019-05-17

**Authors:** Ming-Lung Chuang, Meng-Jer Hsieh, Tzu-Chin Wu, I-Feng Lin

**Affiliations:** 1 Division of Pulmonary Medicine and Department of Internal Medicine, Chung Shan Medical University Hospital, Taichung, 40201 ROC Taiwan; 2 School of Medicine, Chung Shan Medical University, Taichung, 40201 ROC Taiwan; 3 Department of Pulmonary and Critical Care Medicine, Chiayi Chang-Gung Memorial Hospital, Chang-Gung Medical Foundation, Chiayi, ROC Taiwan; 4 Department of Respiratory Therapy, Chang Gung University, Taoyuan, ROC Taiwan; 5 Institute of Public Health, National Yang Ming University, Taipei, ROC Taiwan

**Keywords:** Respiration, Chronic obstructive pulmonary disease, Diagnostic markers

## Abstract

Tidal volume at peak exercise and vital capacity ratio (V_Tpeak_/VC) and V_Tpeak_/inspiratory capacity (IC) were used to differentiate lung expansion in subjects with normal health and chronic obstructive pulmonary disease (COPD) from that in subjects with restrictive ventilation. However, VC and IC variably change due to pseudorestriction of lung volumes. Thus, these variables are currently not recommended. In contrast, total lung capacity (TLC) does little change during exercise. The aims of the study investigated whether V_Tpeak_/TLC is more significantly correlated with static air trapping and lung hyperinflation in patients with COPD than V_Tpeak_/IC, V_Tpeak_/FVC, and V_Tpeak_/SVC (study 1), and developed a marker to replace dynamic IC maneuvers by evaluation of the relationship between end-expiratory lung volume (EELV) and V_Tpeak_/TLC and identification of a cutoff value for V_Tpeak_/TLC (study 2). One hundred adults with COPD (study 1) and 23 with COPD and 19 controls (study 2) were analyzed. Spirometry, lung volume, diffusing capacity, incremental cardiopulmonary exercise tests with dynamic IC maneuvers were compared between groups. An ROC curve was generated to identify a cut off value for V_Tpeak_/TLC. In study 1, V_Tpeak_/TLC was more significantly associated with airflow obstruction, static air trapping and hyperinflation. In study 2, V_Tpeak_/TLC was highly correlated with EELV in the patients (r = −0.83), and V_Tpeak_/TLC ≥ 0.27 predicted that 18% of the patients with static air trapping and hyperinflation can expand their V_T_ equivalent to the controls. In conclusions, V_Tpeak_/TLC was superior to other V_Tpeak_/capacities. V_Tpeak_/TLC may be a marker of dynamic hyperinflation in subjects with COPD, thereby avoiding the need for dynamic IC maneuvers. V_Tpeak_/TLC < 0.27 identified approximately 82% of subjects with COPD who could not adequately expand their tidal volume. As most of our participants were male, further studies are required to elucidate whether the results of this study can be applied to female patients with COPD.

## Introduction

In normal healthy subjects, tidal volume (V_T_) rapidly expands at the start of rapid incremental exercise and gradually reaches a plateau after reaching approximately 55% of vital capacity (VC)^1^. V_T_/VC and V_T_ and inspiratory capacity ratio (V_T_/IC) have been reported to range from 0.55 ± 0.09 to 0.63 ± 0.19 and from ≤0.7 ± 0.11 to 0.79 ± 0.04^1–6^, respectively. V_T_/IC values in subjects with restrictive ventilatory impairment usually reach 1, up to the limit of IC^1^.

Reduced dynamic inspiratory reserve volume (IRV) or O’Donnell’s threshold^7^ combined with elevated end-expiratory lung volume (EELV) can substantially constrain expansion of operating V_T_ in patients with chronic obstructive pulmonary disease (COPD). Furthermore, V_T_/VC tends to be more variable because of variations in SVC and FVC in subjects with COPD due to pseudorestriction (i.e., low FVC%pred but normal total lung capacity, TLC). In this context, variations in operating V_T_ at peak exercise and the “false” restriction of FVC (or SVC or IC) lead to inconsistent values of V_T_/FVC, V_T_/SVC, and V_T_/IC. Therefore, these variables are not recommended to differentiate obstructive from restrictive ventilatory limitations^8^.

Dynamic IC measurements have been reported to be a good way to identify dynamic hyperinflation^2–5,7^. However, IC maneuvers have to be standardized and the study subjects have to become familiarized with the maneuvers, and IC measurements and analysis also have to be standardized by researchers^9^. Nevertheless, dynamic IC measurements are not recommended for ramp-pattern protocols in which V_T_ cannot steadily proceed to perform IC maneuvers^9^. However, the ramp-pattern protocol is a widely used protocol to test incremental exercise.

TLC does not change or only changes a little during exercise in normal subjects and subjects with lung diseases^10–12^. We hypothesized that V_T_/TLC at peak exercise (V_Tpeak_/TLC) would be lower in subjects with COPD compared to normal subjects, and that it would be less variable than V_Tpeak_/FVC, /SVC and /IC. As TLC and V_Tpeak_ are routinely measured during lung function tests and cardiopulmonary exercise tests (CPET), respectively, V_Tpeak_/TLC may be a convenient new marker of dynamic hyperinflation, thereby avoiding the need for dynamic IC measurements.

The aims of this study were (1) to investigate whether V_Tpeak_/TLC is less variable and more significant in correlation with static air trapping or lung hyperinflation in subjects with COPD than V_Tpeak_/IC, /FVC, and /SVC, and (2) to develop a new marker of dynamic hyperinflation to replace dynamic IC maneuvers by evaluation of the relationship between EELV and V_Tpeak_/TLC and identification of a cutoff value of V_Tpeak_/TLC.

## Methods

### Study design

This observational cross-sectional study enrolled healthy normal subjects and subjects with COPD at two university teaching hospitals, and analyzed lung function and cardiopulmonary exercise data for *aim 1* and *aim 2*. The Institutional Review Boards of Chung Shan Medical University Hospital (CS16174) and Chang Gung Memorial Hospital (201700899A3) approved this study, which was conducted in compliance with the Declaration of Helsinki.

### Subjects

Subjects aged ≥40 years without any chronic diseases including uncontrolled diabetes mellitus, uncontrolled hypertension, anemia (hemoglobin <13 g·dL^−1^ in males and <12 g·dL^−1^ in females), and no acute illnesses in the recent 1 month were enrolled. Anthropometric measurements, leisure/sports activities, and cigarette smoking were recorded. Subjects with a body mass index ≤18 kg·m^−2^ or ≥32 kg·m^−2^ or with laboratory findings of cardiovascular, hematological, metabolic or neuromuscular diseases were excluded.

#### Study group

**Figure 1:**
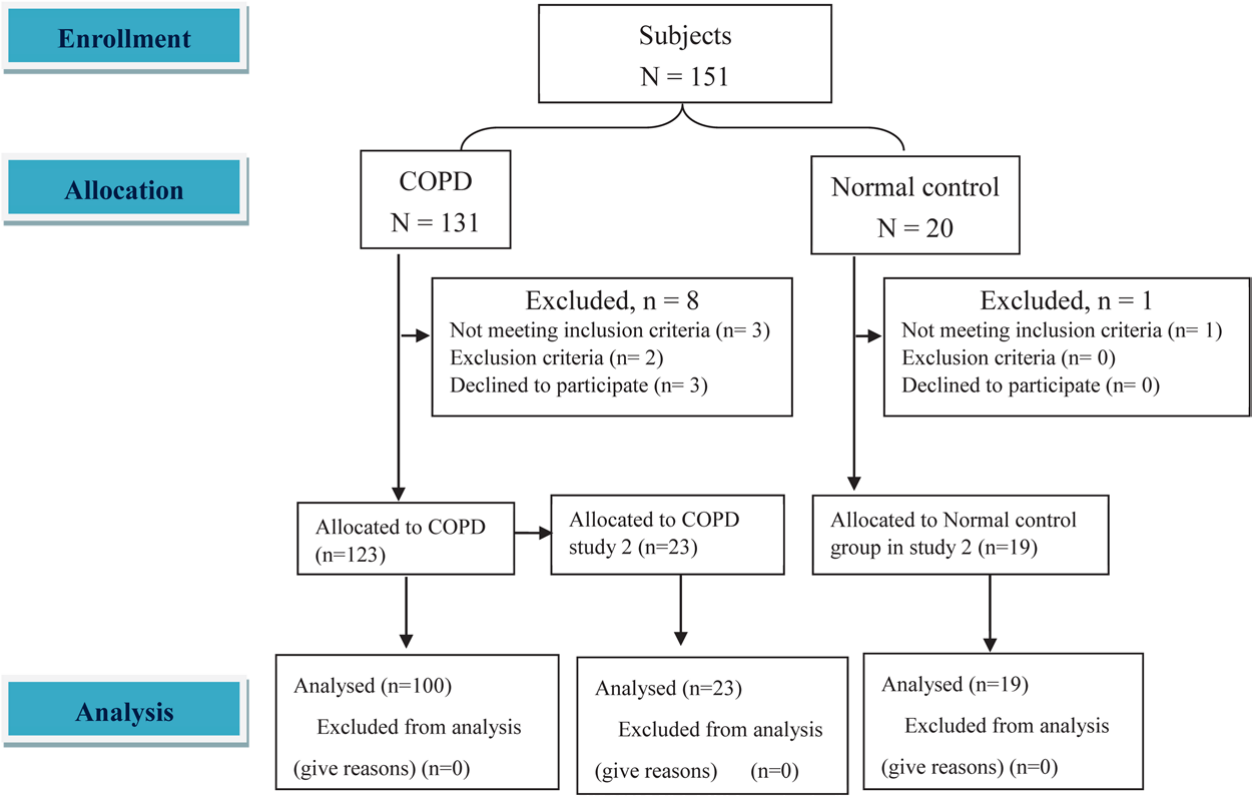
Flow diagram. A total of 131 subjects with chronic obstructive pulmonary disease were screened along with 20 normal healthy controls. After excluding eight subjects with COPD, the remaining 123 were allocated to study 1 (n = 100) and study 2 (n = 23). After excluding one subject, the remaining 19 normal subjects were allocated to study 2.

COPD was diagnosed according to the GOLD criteria^13^. Adult subjects who underwent lung function tests were enrolled only if their FEV_1_/FVC was <0.7 or the flow volume curve of spirometry revealed typical concavity^13^, and their forced expired volume in one second (FEV_1_)% predicted was <80% and had been stable for at least 1 month. A total of 131 subjects in the study group were screened, and 123 were retained for the study (Fig. 1). The reasons for exclusion included not meeting the inclusion criteria (n = 3), meeting the exclusion criteria (n = 2), and declining to participate (n = 2). The study group was divided into two cohorts, one for *aim 1* and one for *aim 2*. To evaluate the bronchodilator effect on the relationship between V_Tpeak_/TLC and static air trapping or lung hyperinflation, the subjects with COPD in study 1 were not allowed to use medications before measurements, whereas the subjects with COPD in study 2 were used medications as normal.

#### Control group

Healthy subjects without any of the aforementioned chronic diseases were screened. Twenty such subjects were screened, and 19 were enrolled (one subject did not meet the inclusion criteria) (Fig. 1). All eligible subjects were enrolled after signing informed consent forms.

### Definitions

#### Static and dynamic air trapping and hyperinflation

Definitions of pulmonary hyperinflation and air trapping of the lung in the literature are inconsistent. In this study, static air trapping and hyperinflation were defined as RV/TLC > 0.45^14,15^, FRC%pred > 120%, or RV%pred > 120%; dynamic air trapping or hyperinflation was defined as V_Tpeak_/TLC < 0.4^2,3,5^.

### Measurements

#### Pulmonary function testing

FEV_1_, TLC, RV, and diffusing capacity for carbon monoxide were measured using spirometry and body plethysmography (6200 Autobox DL, Yorba Linda, CA, USA or MasterScreen Body™, Carefusion, Wuerzburg, Germany) according to recommended standards^16–21^. V_T_/TLC, V_T_/SVC, V_T_/FVC, and V_T_/IC were derived when V_T_ was obtained at peak exercise.

#### CPET

Each subject completed an incremental exercise test to the limit of the symptom (MasterScreen CPX™, Carefusion, Wuerzburg, Germany). Work rate was selected at a rate of 5–20 W/min based on a derived protocol formula according to the oxygen-cost diagram scores^22^. $\dot{{\rm{V}}}$O_2_ (mL/min), CO_2_ output ($\dot{{\rm{V}}}$CO_2_) (mL/min), and minute ventilation ($\dot{{\rm{V}}}$_E_) were continuously measured. $\dot{{\rm{V}}}$O_2peak_ was symptom-limited and $\dot{{\rm{V}}}$O_2peak_ predictions were performed as reported previously^22^. Cardiovascular stress level or exercise intensity was defined as heart rate at peak exercise/heart rate predicted maximum. The definition of ventilatory limitation was a breathing reserve of either <30% or <11–15 L/min^23^.

#### Dynamic inspiratory capacity measurement

The techniques used for performing and accepting IC measurements were as previously reported^9^. Dynamic IC was measured at the end of a steady-state resting baseline and unloaded cycling, and near the middle of loaded exercise and near end exercise. The middle of the loaded exercise was approximately 5–6 minutes after the start of loaded exercise, when dynamic IC near anaerobic threshold was measured. EELV was calculated as TLC minus dynamic IC. O’Donnell threshold was calculated as dynamic IC minus V_T_ at peak exercise^7^.

### Statistical analysis

Data were summarized as mean ± standard deviation and percentage and 5^th^ and 95^th^ percentiles. The Student’s *t* test was used for comparisons between two groups. Correlations were based on Pearson’s correlation coefficients. A *p* value < 0.05 was considered to be significant. An ROC curve was generated to identify a cut off value for V_Tpeak_/TLC by comparisons with dynamic EELV at peak exercise. Statistical analyses were performed using SAS statistical software (SAS Institute Inc., Cary, NC, USA) and Origin v4.1 (Northampton, MA, USA). The sample size of study 2 was estimated to be 23 based on calculations with a 0.1 between-group difference and 0.1 of standard deviation for each group in V_Tpeak_/TLC with a power of 0.9 and significance level of 0.05.

## Results

One hundred subjects (97 men) with COPD in study 1 and 23 (23 men) with COPD and 19 healthy subjects (19 men) in study 2 were analyzed (Fig. 1 and Table 1). The majority of the subjects in study 1 had moderate airflow obstruction with hyperinflation and air trapping, and mild exercise hyperventilation and exercise ventilation limitation with mild exercise impairment (Tables 1 and 2). Compared to study 1, most of the subjects with COPD in study 2 had less severe airflow obstruction and hyperinflation and exercise impairment (Tables 1 and 2). V_Tpeak_/TLC and V_Tpeak_/SVC were significantly lower in the subjects in study 1 than in the COPD group in study 2, while V_Tpeak_/FVC and V_Tpeak_/IC were similar between the two COPD groups.

**Table 1:** Demographics and lung function in 100 subjects with chronic obstructive pulmonary disease (COPD) in Study 1 and another 23 subjects with COPD and 19healthy subjects in Study 2.

	COPD1		COPD2		Normal		P	
	mean	SD	mean	SD	mean	SD	COPD Study 1 vs 2	Study 2 COPD vs normal
N=	100		23		19			
Age, years	67.8	7.6	68.2	7.7	64.9	9.9	0.93	0.22
Sex: M:F	97:3		23:0		20:0			
Height, cm	164.2	6.1	164.8	5	167.1	5.2	0.63	0.14
Weight, kg	61.1	9.6	61.1	10.7	70.5	8.5	0.91	0.003
Body mass index, kg/m^2^	22.7	3.1	22.5	3.7	25.5	2.7	0.69	0.01
Cigarette smoke, pack × year	46	22.8	69.2	38.5	37.8	40.8	0.0001	0.14
Total lung capacity, TLC, L	6.1^#^	1	6.25	0.89	5.88	0.81	0.62	0.16
%predicted, %	120	23	115	17	104	13	0.282	0.03
FRC, L	4.4^#^	1	4.19	1.06	3.14	0.51	0.47	0.0002
%predicted, %	141	35	138	34	102	16	0.69	0.0003
FRC/TLC	0.71^#^	0.09	0.67	0.1	0.53	0.05	0.04	<0.0001
Residual volume, RV, L	3.5^#^	1	3.6	1.1	2.42	0.55	0.61	0.0001
%predicted, %	168	54	166	48	112	22	0.83	<0.0001
RV/TLC	0.56^#^	0.1	0.57	0.11	0.41	0.07	0.77	<0.0001
Inspiratory capacity, IC, L	1.78^#^	0.55	2.01	0.54	2.75	0.48	0.06	<0.0001
%predicted, %	88	27	82	21	108	19	0.29	0.0004
IC_dynamic_, L	—	—	2.22^∧^	0.66	3.03^∧^	0.69	—	0.003
IC/TLC	0.29^#^	0.09	0.33	0.09	0.47	0.05	0.09	<0.0001
D_L_CO, mL/mm Hg/min	14.5	5.2	13.3	5.1	19.2	3.2	0.26	<0.0001
%predicted, %	75^#^	27	78	21	102	16	0.66	0.0001
Forced vital capacity, FVC, L	2.51	0.65	2.62	0.66	3.45	0.58	0.45	<0.0001
%predicted, %	83	22	83	14	101	13	0.99	0.0002
FEV_1_, L	1.29	0.46	1.53	0.54	2.76	0.47	0.02	<0.0001
%predicted, %	55	19	64	16	104	12	0.03	<0.0001
FEV_1_/FVC	0.52	0.12	0.57	0.11	0.8	0.05	0.02	<0.0001
Slow vital capacity, SVC, L	2.7^&^	0.66	2.66	0.64	3.46	0.57	0.88	<0.0001
%predicted, %	88	24	80	14	99	13	0.11	<0.0001
MVV, L/min	43	17.8	63.5	22.4	108.6	21.1	<0.0001	<0.0001

Functional residual capacity, FRC; Inspiratory capacity, IC; Diffusing capacity for carbon monoxide, DLCO; Forced expired volume in one second, FEV_1_; MVV. Maximum voluntary ventilation,GOLD I, n = 10; GOLD II, n = 49; GOLD III, n = 32; GOLD IV, n = 9. ^#^n = 97, ICdyn: IC dynamic, measured during incremental exercise testing, ^&^n = 99, *Study 1vs2 on subjects with COPD; ^∧^p = 0.12 and 0.14, respectively, intra-group comparison.

**Table 2:** Cardiopulmonary exercise test at peak exercise in 100 subjects with chronic obstructive pulmonary disease (COPD) in Study 1 and another 19 healthy subjects and 23subjects with COPD in Study 2.

	COPD1		COPD2		Normal		P	
	mean	SD	mean	SD	mean	SD	Study1 vs 2 COPD	Study2 COPD vs normal
N=	100		23		19			
Work rate, watts	83.2	36.3	95.9	38.5	141.4	33.9	0.14	0.0002
% pred	72	28	85	25	113	25	0.04	0.0008
Oxygen uptake (VO_2_), l/min	1.03	0.33	1.27	0.47	1.56	0.37	0.005	0.03
% pred	67	20	74	20	84	14	0.18	0.06
Anaerobic threshold, l/min	0.67	0.18	0.82	0.23	0.99	0.32	0.002	0.07
%VO_2max_pred, %	44	13	48	13	53	12	0.18	0.28
Respiratory exchange ratio	1.05	0.11	1.03	0.1	1.17	0.11	0.46	0.0001
Cardiac frequency, b/min	128	19.5	127.7	19.3	147.9	18.9	0.97	0.001
% pred max, %	82	12	84	11	95	11	0.49	0.001
Oxygen pulse, mL/min	8.1	2.4	9.7	2.7	10.6	2	0.004	0.27
% pred	82	23	87	18	88	15	0.36	0.8
Minute ventilation (V_E_)/VO_2nadir_	39.7	7.7	32.4	6.8	29.6	3.9	0.0002	0.12
S_P_O_2_,%	92	5	92	8	97	1	0.71	0.02
V_E_, l/min	41.5	12.2	49.3	16.7	65.6	16.4	0.01	0.003
VE/MVV	1.03	0.31	0.82	0.1	0.59	0.11	0.002	<0.0001
Breathing frequency, b/min	33.4	5.8	33	7.5	33.6	5.7	0.77	0.77
Tidal volume (V_T_), L	1.25	0.35	1.48	0.35	1.97	0.41	0.005	0.0002
V_T_/TLC	0.21^#^	0.06	0.24	0.07	0.33	0.05	0.01	<0.0001
V_T_/SVC	0.48^&^	0.13	0.56	0.12	0.57	0.1	0.004	0.76
V_T_/FVC	0.51	0.16	0.57	0.12	0.57	0.1	0.11	0.98
V_T_/inspiratory capacity (IC)	0.74^#^	0.21	0.77	0.14	0.72	0.13	0.55	0.33
V_T_/dynamic IC	—	—	0.71^∧^	0.13	0.65^∧^	0.14	—	0.28
EELV @ rest, L	—	—	3.34	2.46	3	0.9	—	0.75
EELV @ unloading, L	—	—	3.54	2.0	2.70	1	—	0.17
EELV @ AT, L	—	—	3.37	2.1	2.68	0.9	—	0.27
EELV @ peak, L	—	—	4.17	1.29	2.75	0.8	—	0.001

Maximum voluntary ventilation, MVV; TLC, total lung capacity; SVC or FVC, slow or forced vital capacity; EELV, end-expiratory lung volume; ^#^n = 97, ^&^n = 99, ^∧^Intra-group comparison of V_T_/IC and V_T_/dynamic IC, p = 0.12 and 0.11, respectively.

### Correlation of V_Tpeak_/TLC, /SVC, /FVC, and /IC with static air trapping and spirometry

V_Tpeak_/TLC was 0.21 ± 0.06 (Table 2) and was most significantly correlated with air trapping or hyperinflation parameters and FEV_1_/FVC and FEV_1_%pred compared to the other three variables (Table 3, |r| = 0.45–0.62 vs. 0.001–0.49). Twelve of 99 (12%) subjects had a V_Tpeak_/SVC > 0.63 (mean cut-off value of normal and COPD groups ≤0.63^1,4,5^), 18 of 100 subjects had a V_Tpeak_/FVC > 0.63, and 31 of 97 (32%) subjects had a V_Tpeak_/IC > 0.79 (cut-off value: ≤0.79^1–3,5^), while none of 97 subjects had a V_Tpeak_/TLC > 0.4 (re-measured normal value: 0.4–0.42^2,3,5^, Table 4) and only two (2%) had a V_Tpeak_/TLC > 0.33 (Table 2, normal cut-off value in study 2).

**Table 3:** Correlation coefficients of the ratios of tidal volume at peak exercise (V_Tpeak_) and TLC, SVC, FVC, and IC with markers of air trapping or lung hyperinflation and spirometry at rest in subjects with chronic obstructive pulmonary disease.

Study 1, n = 100	V_Tpeak_/TLC	V_Tpeak_/SVC	V_Tpeak_/FVC	V_Tpeak_/IC
FRC/TLC	−0.53^‡^	0.001	−0.09	0.49^‡^
RV/TLC	−0.59^‡^	0.21^¶^	0.14	0.08
FRC%pred	−0.50^‡^	−0.05	−0.03	0.26^*^
RV%pred	−0.61^‡^	0.01	0.002	0.04
FEV_1_/FVC	0.45^‡^	0.26^*^	0.31^†^	0.15
FEV_1_%pred	0.62^‡^	0.02	−0.06	0.11
**Study 2, n** = **23**				
FRC/TLC	−0.73^‡^	0.001	0.13	0.52*
RV/TLC	−0.67^†^	0.28	0.39	0.36
FRC%pred	−0.77^‡^	−0.03	0.11	0.22
RV%pred	−0.65^†^	0.12	0.24	0.14
FEV_1_/FVC	0.76^‡^	0.45^*^	0.33	−0.01
FEV_1_%pred	0.79^‡^	0.15	0.01	−0.16

Forabbreviations, please refer to Table 1. ^*^p < 0.05, ^†^<0.01, ^†^<0^.^001, ^‡^<0.0001, ^¶^<0^.^1.

**Table 4:** Summary from this study and the literature regarding the ratio of operating tidal volume at peak exercise and total lung capacity (VTpeak/TLC) and other relevant ratios in subjects with chronic obstructive pulmonary disease (COPD) and subjects with interstitial lung disease (ILD) and normal healthy subjects.

	COPD						Normal				
	This study1, 2	IPO^26^ (−)	IPO^26^ (+)	O’Donnell 1993, 2001	Ciavaglia^27^, obese	Faisal^2^, obese	This study2	Spiro^6^	O’Donnell^3–5^	Amann^28^	Faisal
N=	100, 23	33	14	23, 105	12	16	19	20	10, 12, 25	5	16
V_Tpeak_/TLC, mean	0.21^∧^–0.24	0.2^∧^	0.14^∧^	0.15–0.17	0.24	0.24	0.31	—	0.4–0.42	0.3	0.41
5%tile	0.11–0.14	0.13	0.09	—	—	—	0.23	—	—		—
95%tile	0.3–0.36	0.27	0.24	—	—	—	0.42	—	—		—
V_Tpeak_/VC, mean	0.48*–0.56	0.48^∧^	0.37*	0.44–0.6	0.46	0.46	0.57	0.58	0.61–0.66		0.56
5%tile	0.3–0.39	0.31	0.24	—	—	—	0.39	—	—		—
95%tile	0.72–0.8	0.72	0.52	—	—	—	0.70	—	—		—
V_Tpeak_/VC_dyn_, mean (SD)	—	—	—	0.31 (0.1)	—	—	—	—	0.63 (0.19)		—
V_Tpeak_/IC, mean	0.74–0.77	0.78	0.61*	0.65	0.56	0.66	0.72	—	0.78		0.76
5%tile	0.47–0.57	0.5	0.40	—	—	—	0.49	—	—		—
95%tile	1.08–0.92	1.05	0.78	—	—	—	0.86	—	—		—
V_Tpeak_/IC_dyn_, mean (SD)	0.71 (0.13)	—	—	0.74 (0.14)	0.77	0.76 (0.13)	0.65 (0.14)	—	0.74–0.78 (0.04^SE^–0.15)		0.78 (0.08)

IPO^26^: impaired peripheral oxygenation. ^∧^P < 0.0001 vs the normal subjects of the study 2, *P = 0.02 vs study 2 norm, ^SE^Standard error.

### Relationship between V_Tpeak_/TLC and static air trapping and airflow obstruction

**Figure 2:**
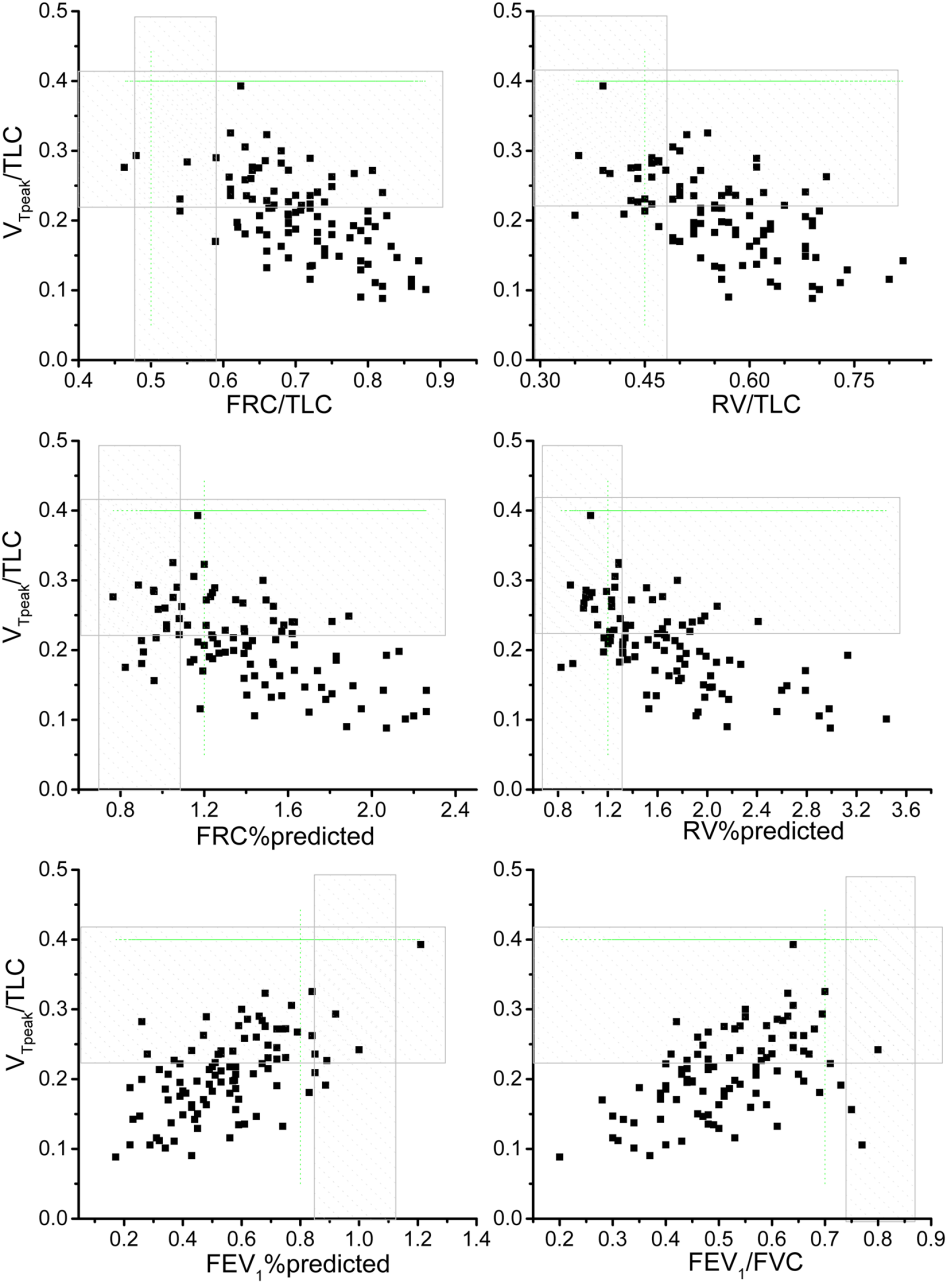
Tidal volume at peak exercise (V_Tpeak_) and total lung capacity (TLC) ratio as a function of air trapping, hyperinflation and airflow obstruction in the subjects with COPD (n = 97). The hatched areas represent the 5^th^ and 95^th^ percentiles of each corresponding variable of the normal subjects in study 2. The vertical and horizontal dashed lines represent the mean value of each corresponding variable of the normal subjects from the literature. Each symbol represents one subject.

The normal subjects could expand V_Tpeak_/TLC from 0.25 to 0.41 during incremental exercise (Fig. 2). The area under ROC curve was 0.861 and V_Tpeak_/TLC of 0.27 had the highest sensitivity (88%) and specificity (77%) to predict elevated EELV. Nineteen of 112 (17%) subjects with FRC/TLC > 0.59 (Table 1, 95^th^ percentile of the normal subjects in study 2) could expand their lungs to ≥ V_Tpeak_/TLC of 0.27. Sixteen 13 of 97 (13%) subjects with RV/TLC > 0.48 (95^th^ percentile of normal), 28of 119 (24%) subjects with FEV_1_%pred < 88% (5^th^ percentile of normal), and 21 of 120 (18%) subjects with FEV_1_/FVC < 0.73 (5^th^ percentile of normal) could expand their lungs to ≥V_Tpeak_/TLC of 0.27.

### Correlation of V_Tpeak_/TLC and EELV or other variables

**Figure 3:**
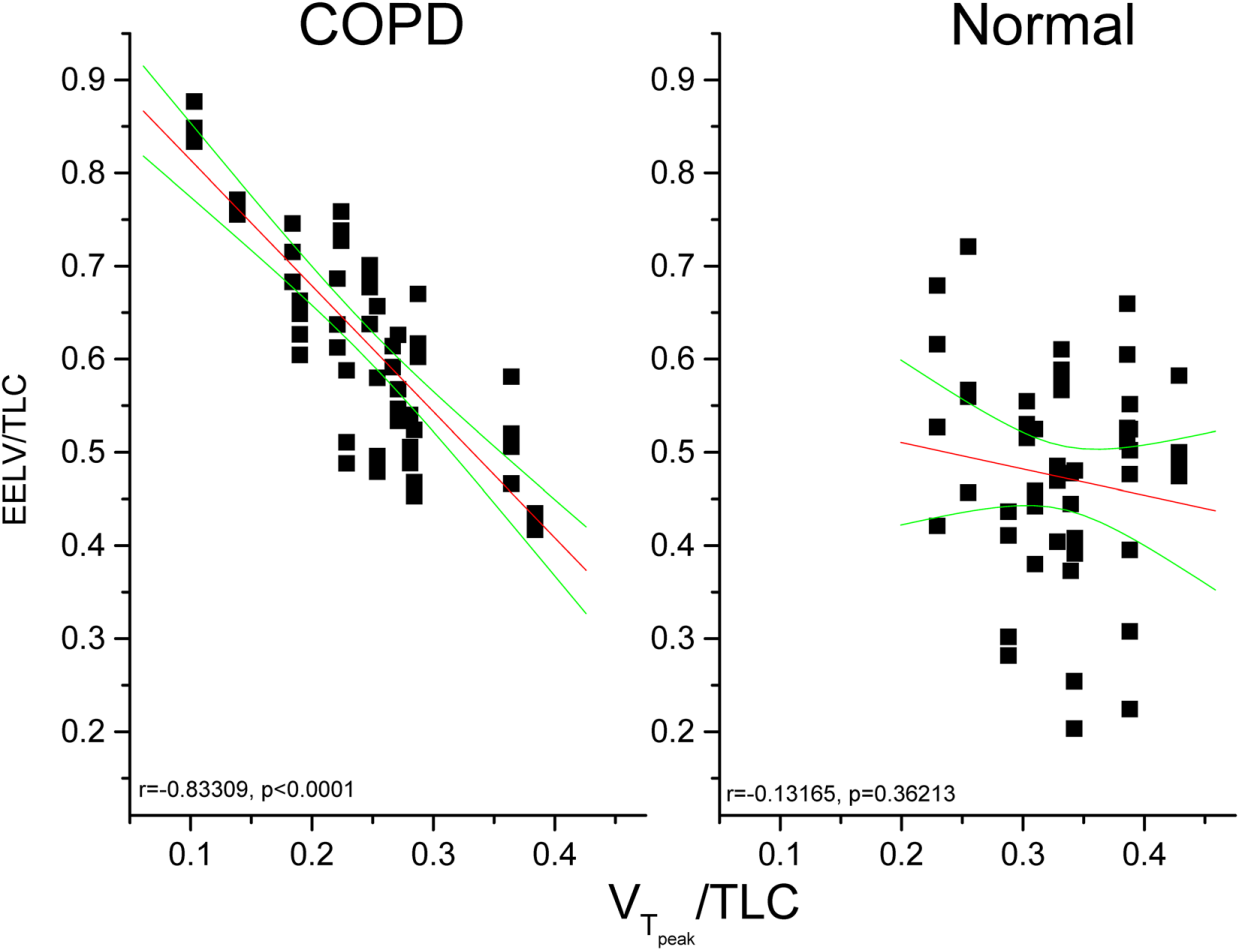
End-expiratory lung volume and total lung capacity ratio (EELV/TLC) at rest, unloading, near anaerobic threshold, and near peak exercise as a function of tidal volume at peak exercise and TLC ratio (V_Tpeak_/TLC) in 23 subjects with COPD and 19 normal subjects. The solid line represents the linear regression line (r = −0.83, p < 0.0001 for the COPD group and r = −0.13, p = 0.36 for the normal subjects) and the curved lines are the 95% CI lines.

In study 2, the levels of EELV at rest, unloading exercise, and near anaerobic threshold and near peak exercise were significantly larger in the subjects with COPD than in the normal subjects (Table 2). V_Tpeak_/TLC was significantly correlated with EELV/TLC in the subjects with COPD but not in the normal subjects (Fig. 3, r = −0.83, p < 0.0001 vs. r = −0.13, p = 0.36). V_Tpeak_/TLC was also significantly correlated with $\dot{{\rm{V}}}$O_2peak_% (r = 0.73, p < 0.0001) in the COPD group but not in the normal group (r = 0.24, p = 0.33). However, V_Tpeak_/TLC was not significantly correlated with Borg score (r = −0.03 vs. −0.03, both p = NS) or O’Donnell threshold (r = −0.43 vs. 0.2, both p = NS) in either groups.

## Discussion

The key findings of this study were that V_Tpeak_/TLC was the best marker for dynamic expandability of lungs compared to V_Tpeak_/SVC, V_Tpeak_/FVC, and V_Tpeak_/IC in the subjects with COPD (Table 3, |r| = 0.45–0.62 vs. 0.001–0.49). These findings were noted in the subjects with COPD with or without withdrawal of bronchodilators before the test (Table 3 in study 2, |r| = 0.65–0.79 vs. 0.001–0.52). V_Tpeak_/TLC was highly correlated with EELV in the subjects with COPD but not in the normal subjects (Fig. 3, r = −0.83 vs. −0.13). A V_Tpeak_/TLC cutoff value of <0.27 further identified approximately 82% of the subjects with airflow obstruction and static air trapping or hyperinflation who developed dynamic air trapping during incremental exercise (Fig. 2). As V_Tpeak_/TLC can easily be obtained without the need for any maneuvers during exercise, the need for dynamic IC maneuvers during exercise test may be avoided.

### V_Tpeak_/TLC, /SVC, /FVC, and /IC

V_Tpeak_/SVC and /IC have been used to differentiate normal and obstructive ventilation from restrictive ventilation during CPET^24,25^. V_Tpeak_/FVC has also been used as an alternative due to the ease of obtaining FVC. However, these variables are not recommended by the American Thoracic Society for such purposes^8^. In this study, V_Tpeak_/SVC, /FVC, and /IC showed much higher variability than V_Tpeak_/TLC in the correlation with static air trapping (Table 3). This may be because TLC changes little during exercise in normal subjects and those with airflow obstruction and interstitial lung disease^10–12^. As dynamic air trapping develops, V_T_ is restricted and air cannot be removed sufficiently, thereby limiting the increase in V_Tpeak_/TLC. In contrast, static SVC, FVC, and IC may be variable due to pseudorestriction of lung volume even in subjects with the same TLC, so that dynamic hyperinflation cannot be precisely predicted by V_Tpeak_/SVC, /FVC, and /IC. Pseudorestriction is not uncommon in subjects with airflow obstruction, small airway obstruction and emphysema.

O’Donnell *et al*. reported that changes in dynamic IC were larger (−14%) during exercise in subjects with airflow obstruction than in normal subjects (4%, p < 0.0005)^4^, meaning that V_Tpeak_/dynamic IC was larger than V_Tpeak_/static IC (74% vs. 65%)^4^. However, in the current study, V_Tpeak_/dynamic IC was smaller but not significantly smaller than V_Tpeak_/static IC (71% vs. 77%) in the subjects with COPD, suggesting that dynamic IC was larger than static IC. This was also noted in the normal subjects in study 2 (i.e., 0.72 vs. 0.65; Table 2). This may be due to different severities of COPD in these two studies. In O’Donnell’s study^4^, the patients with COPD had 37%pred FEV_1_ in contrast to study 2 where the subjects with COPD had 55–68%pred FEV_1_.

### V_Tpeak_/TLC ratio and EELV or other variables

EELV is calculated as TLC minus dynamic IC. As TLC changes little during exercise^10–12^, changes in EELV must be inversely associated with changes in IC^4^. As mentioned, V_Tpeak_/TLC changed in line with dynamic IC, and was thus inversely related to EELV (Fig. 4, r = −0.83, p < 0.0001). The area under ROC curve was high (0.861) and the sensitivity (88%) and specificity (77%) of VT_peak_/TLC of 0.27 to predict elevated EELV in the current study were acceptable. The importance of V_Tpeak_/TLC has not previously been addressed or reported, and our findings seem to suggest that V_Tpeak_/TLC could be used as a substitute for dynamic IC maneuvers performed during exercise. One reason is that V_Tpeak_/TLC is easily obtainable, and another is that in the ramp pattern exercise protocol but not a steady state protocol, dynamic IC maneuvers are not recommended^9^. Moreover, dynamic V_T_ represents the difference between dynamic end-inspiratory lung volume (EILV) and EELV, and while measuring V_Tpeak_/TLC is straightforward, measuring EILV and EELV is more complex^5^. Interestingly, in the current study V_Tpeak_/TLC was correlated with $\dot{{\rm{V}}}$O_2peak_ in the COPD group rather than in the normal group. This suggests that dynamic lung expansion played a role in the exercise capacity in patients with COPD but not in health. However, both groups reached a similar level of Borg dyspnea score (COPD vs. normal, 6 ± 3 vs. 6 ± 2, p = 0.83) but different levels of O’Donnell threshold/TLC (COPD vs. normal, 0.11 ± 0.07 vs. 0.19 ± 0.12, p = 0.02) at peak exercise. However, V_Tpeak_/TLC was not correlated with either variable. We speculate that Borg dyspnea score and O’Donnell threshold are more related to the plateau portion of the pressure-volume curve for the lungs and chest wall whereas V_Tpeak_/TLC involves lung volumes not only expanding to the plateau portion of the curve but also encroaching downward to expiratory reserve volume.

### V_Tpeak_/TLC ratio in this study and previous reports

Table 4 reveals that V_Tpeak_/TLC was 0.21 ± 0.06–0.24 ± 0.07 in study 1 and 2, which is larger than in our previous report on patients with COPD (0.14–0.2)^26^ and reports on COPD from other researchers (0.15–0.24, the values were not reported in their studies but were re-calculated by the current authors)^2,5,27,28^. This may be due to differences in the severity of COPD and preconditioning strategy before the measurements in these studies. We re-measured V_Tpeak_/TLC from the figures of the previous studies, and found values of 0.31–0.42 in patients with interstitial lung disease^3,10^ and 0.3–0.42 in normal healthy subjects^2,3,5,28^. In the normal subjects of this study, the V_Tpeak_/TLC was 0.31 ± 0.06. However, it is difficult to compare this value between our study and studies in the literature as the level of V_Tpeak_/TLC has not been reported.

### Study limitations

The normal subjects had significant cigarette consumption despite having normal spirometry. In addition, subjects with restrictive ventilation were not included as interstitial lung disease is rare in our institutions. However, V_Tpeak_/TLC in patients with interstitial lung disease as re-measured from the figures of previous studies was 0.31–0.42^2,3,10^. These values are quite different from those reported in the subjects with COPD in this study and in the literature. The number of participants in study 2 was small and the findings may not be generalizable to all populations. However, the sample size of study 2 was estimated to be 23 based on standard calculations. The cohort was all men because the incidence of COPD in women is very low in Taiwan (37:1 in our previous report^29^). Dynamic IC measurements are recommended for subjects who can achieve a steady state of exercise. The exercise protocol in this study was the ramp pattern, and the relationship between V_Tpeak_/TLC and EELV may be different between two-minute incremental and ramp-pattern exercise. However, it can be difficult to reach a steady state in each stage of exercise despite using the two-minute incremental exercise protocol^2–5^. Lastly, the subjects did not undergo pre-test exercise testing including dynamic IC maneuvers to allow them to become familiar with the whole protocol. However, this may more accurately reflect cardiopulmonary exercise testing in the real world.

## Conclusion

V_Tpeak_/TLC may be a potential marker of dynamic hyperinflation in subjects with COPD, and its use may avoid the need for dynamic IC maneuvers during incremental exercise. This marker is simple to derive and more stable than other V_Tpeak_-capacity ratios regarding the relationship with static air trapping or hyperinflation, and it was significantly associated with EELV. A cut-off value of V_Tpeak_/TLC < 0.27 identified approximately 82% of the subjects with COPD who had static hyperinflation and air trapping but could not expand their tidal volume to the same extent as the normal subjects. Further large-scale studies are warranted to investigate whether V_Tpeak_/TLC can replace dynamic IC maneuver and whether it can be used to identify the V_Tpeak_/TLC cut-off value. As most of our participants were male, further studies are required to elucidate whether the results of this study can be applied to female patients with COPD.

## Supplementary information

Dataset
